# Quantitative Metabolomics Reveals an Epigenetic Blueprint for Iron Acquisition in Uropathogenic *Escherichia coli*

**DOI:** 10.1371/journal.ppat.1000305

**Published:** 2009-02-20

**Authors:** Jeffrey P. Henderson, Jan R. Crowley, Jerome S. Pinkner, Jennifer N. Walker, Pablo Tsukayama, Walter E. Stamm, Thomas M. Hooton, Scott J. Hultgren

**Affiliations:** 1 Center for Women's Infectious Diseases Research, Washington University School of Medicine, St. Louis, Missouri, United States of America; 2 Division of Infectious Diseases, Department of Internal Medicine, Washington University School of Medicine, St. Louis, Missouri, United States of America; 3 Department of Molecular Microbiology, Washington University School of Medicine, St. Louis, Missouri, United States of America; 4 Department of Internal Medicine, University of Washington, Seattle, Washington, United States of America; 5 Department of Internal Medicine, University of Miami, Miami, Florida, United States of America; Institut Pasteur, France

## Abstract

Bacterial pathogens are frequently distinguished by the presence of acquired genes associated with iron acquisition. The presence of specific siderophore receptor genes, however, does not reliably predict activity of the complex protein assemblies involved in synthesis and transport of these secondary metabolites. Here, we have developed a novel quantitative metabolomic approach based on stable isotope dilution to compare the complement of siderophores produced by *Escherichia coli* strains associated with intestinal colonization or urinary tract disease. Because uropathogenic *E. coli* are believed to reside in the gut microbiome prior to infection, we compared siderophore production between urinary and rectal isolates within individual patients with recurrent UTI. While all strains produced enterobactin, strong preferential expression of the siderophores yersiniabactin and salmochelin was observed among urinary strains. Conventional PCR genotyping of siderophore receptors was often insensitive to these differences. A linearized enterobactin siderophore was also identified as a product of strains with an active salmochelin gene cluster. These findings argue that qualitative and quantitative epi-genetic optimization occurs in the *E. coli* secondary metabolome among human uropathogens. Because the virulence-associated biosynthetic pathways are distinct from those associated with rectal colonization, these results suggest strategies for virulence-targeted therapies.

## Author Summary

Urinary tract infections (UTIs) are among the most common bacterial infections treated by physicians worldwide. Although symptoms of acute infection are often resolved with a course of antibiotics, the same bacterial strain often causes subsequent bouts of symptomatic infection. *Escherichia coli* are the most common bacteria causing UTI and the infecting strains are widely believed to originate from the gastrointestinal tract where multiple *E. coli* strains reside. Here, we use a novel mass spectrometric technique in a population of patients with recurrent UTI to identify how strains that cause UTI differ from other strains that were present in the gastrointestinal tract at the same time. We found that urinary *E. coli* strains preferentially expressed two small molecules called yersiniabactin and salmochelin. These molecules are called *siderophores*, meaning they are able to scavenge iron to support bacterial survival and growth. Synthesis and transport of these small molecules requires a coordinated network of proteins encoded by a collection of different genes. These findings suggest that new antibiotics directed against yersiniabactin or salmochelin-producing *E. coli* strains may be an improved, and more targeted, strategy to prevent recurrent UTIs.

## Introduction

Urinary tract infection (UTI) is a highly prevalent infectious disease with a well-known female predilection and a high incidence of recurrence [1]. *E. coli* is responsible for up to 85% of community-acquired UTI, and previous studies suggest that the same *E. coli* strain can cause recurrent UTI's despite initial antibiotic treatment [2],[3],[4]. UTI has classically been considered to follow inoculation of the bladder through urethral ascension of rectal flora [5]. Urethral ascension to the bladder is considered to be more common in women due to their shorter urethral length and facilitated by mechanical effects on the urethra during intercourse, which is a major risk factor for UTI. The events preceding clinical UTI where colonization progresses to symptomatic bacteriuria are poorly understood and difficult to study.

Whether selection of UTI-associated strains from gut *E. coli* populations is stochastic or the result of intrinsic strain properties has been the subject of multiple investigations. Genes involved with iron acquisition routinely emerge as correlates of urinary pathogenesis in these studies. In one such study, a genome-wide search in the model uropathogen UTI89 revealed extensive selection of 29 genes including those involved in synthesis of the siderophore enterobactin [6]. These siderophore genes were also subject to increased transcription during experimental urinary tract infection [7]. Finally, PCR-based studies of candidate virulence factor genes have identified high frequencies of siderophore receptor genes among urinary isolates although expression of the corresponding siderophores was not determined [8],[9].

Siderophores are a chemically diverse family of small molecules that are produced by a wide variety of pathogenic and non-pathogenic bacteria to scavenge ferric iron (Fe^3+^) [10]. During iron scarcity, these bacteria synthesize and secrete siderophores, which avidly bind ferric iron and become internalized. Siderophores effectively compete with mammalian iron storage proteins and may be of particular importance in acquiring this critical nutrient during infection [11]. Additional horizontally-acquired genes facilitating siderophore biosynthesis may confer new or enhanced properties that may render a bacterium more virulent. To date, genes for various combinations of four genetically distinct siderophore systems have been described in clinical *E. coli* isolates with enterobactin being the only system conserved in all isolates (Table 1). Among the non-conserved siderophores, the synthesis of salmochelin is encoded in the *iroA* gene cluster, involving the IroB-mediated glucosylation and IroE-mediated linearization of enterobactin. The *Yersinia* high pathogenicity island (HPI) encodes the genes necessary for the synthesis and uptake of yersiniabactin. Aerobactin biogenesis is encoded in the *iucABCD* cluster of genes.

**Table 1:** Siderophores described among *E. coli* strains used in this study.

	enterobactin	salmochelin	yersiniabactin	aerobactin
**type**	**catecholate**	**catecholate**	**thiazoline**	**hydroxamate**
synthesis	*entABCDEF* [17],[40]	*iroB*,*E* [19],[28],[41]	*ybtSETU*, *irp1*, *irp2* [18],[42]	*iucABCD* [43]
receptor	*fepA* [44]	*iron* [41]	*fyuA* [45]	*iutA* [43]

In this study we have used a quantitative metabolomics approach together with microbiologic, genomic, and clinical strategies to uncover a preferential metabolic signature among *E. coli* isolates from the urines of women with recurrent UTI (urinary *E. coli*). Comparisons of coincident urinary and rectal strains from patients with recurrent UTI revealed that urinary strains exhibited significantly higher production of yersiniabactin and salmochelin, even amongst genotype-positive strains, but not enterobactin and aerobactin. Also, the siderophore receptor genotype did not always correspond to production of the associated siderophore, in contrast to previous assumptions [8],[12],[13]. Thus, a quantitative metabolomic approach revealed important differences in siderophore production not detectable by genotyping alone. Our analysis of the metabolomic network necessary for siderophore biosynthesis revealed that in addition to its role in salmochelin biogenesis, IroE also converts a conserved siderophore (enterobactin) into a more virulent one (linearized enterobactin) better suited to the infectious microenvironment. These studies demonstrate that *E. coli* strains associated with recurrent urinary tract infection have a preferred metabolomic profile involving a complex metabolic network.

## Results

### The secreted metabolome among clinical *E. coli* isolates contains multiple siderophores

**Figure 1:**
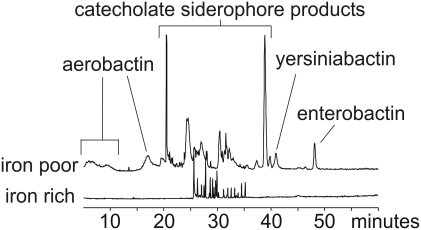
LC-MS metabolite profiling reveals differential product formation based on media iron content. Depicted are total ion chromatograms of conditioned culture media following 18 hour culture of *E. coli* strain rUTI2 in M63 media containing 1 µM (“iron poor”, top trace) or 100 µM ferric chloride (“iron rich”, top trace). Mass spectrometric analysis confirms that the majority of differentially expressed peaks in the iron poor supernatant derive from siderophores.

Siderophore production in 18 previously characterized UPEC strains [14] isolated from the urine of women with UTI was examined. To determine what siderophores are expressed by these *E. coli* isolates, we compared culture supernatants from strains grown for 18 hours in iron-poor and iron-rich minimal media (Fig. 1, Table 2). Comparison of full scan LC-MS profiles from each growth condition revealed a more abundant metabolite signature in iron-poor cultures, consistent with induction of siderophore expression during iron scarcity.

**Table 2:** Siderophore production in previously sequenced or genotyped strains.

strain	enterobactin	salmochelin	yersiniabactin		aerobactin	
	MS	MS	MS	*fyuA*	MS	*iutA*
*MG1655*	+	−	−	−	−	−
*UTI89*	+	+	+	+	−	−
*CFT073*	+	+	**−**	**+**	+	+
*ASB1*	+	+	+	+	−	−
*ASB2*	+	+	+	+	−	−
*ASB3*	+	+	+	+	+	+
*ASB4*	+	−	−	−	−	−
*ASB5*	+	+	+	+	−	−
*acute1*	+	+	+	+	+	+
*acute2*	+	−	−	−	−	−
*acute3*	+	+	+	+	−	−
*acute4*	+	+	+	+	−	−
*rUTI1*	+	−	−	−	−	−
*rUTI2*	+	+	+	+	+	+
*rUTI3*	+	+	+	+	−	−
*rUTI4*	+	−	+	+	**−**	**+**
*rUTI5*	+	+	+	+	+	+
*pyelo1*	+	−	**−**	**+**	−	−
*pyelo2*	+	−	−	−	−	−
*pyelo3*	+	−	**−**	**+**	**−**	**+**
*pyelo4*	+	+	+	+	+	+

The presence or absence of siderophore production as determined by mass spectrometry (MS) is indicated with the presence or absence of the corresponding siderophore receptor genotype.

**Figure 2:**
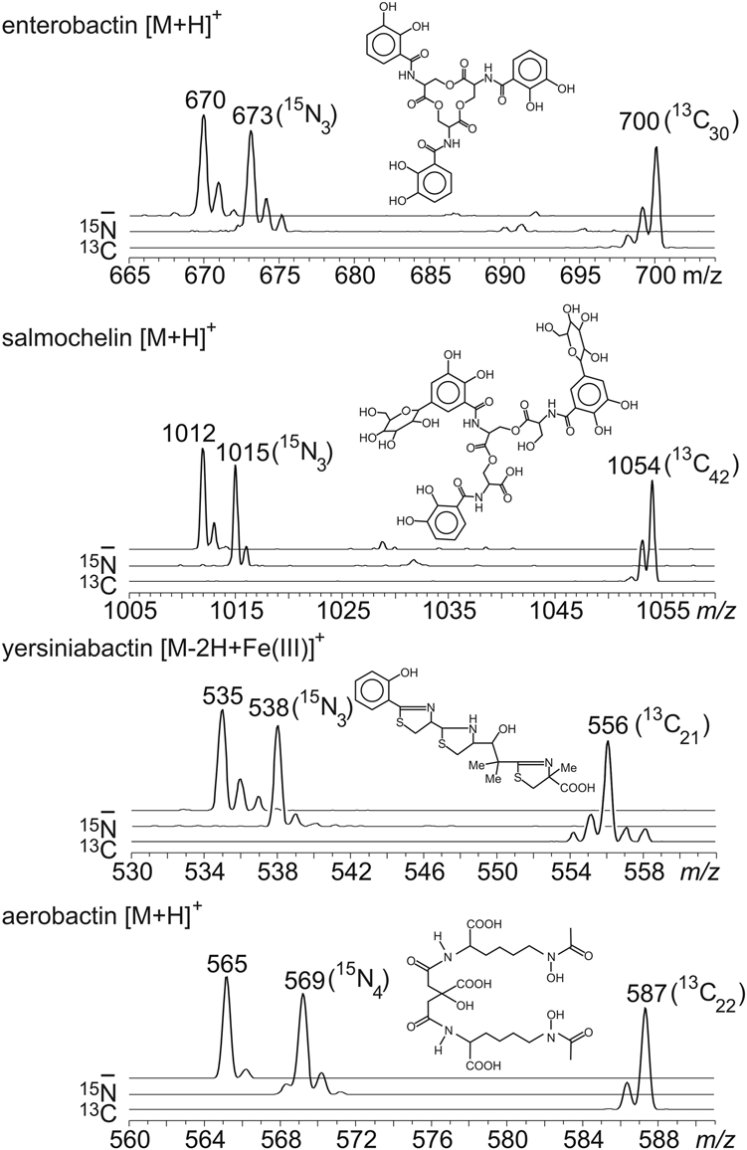
Identity of *E. coli* siderophore peaks is confirmed by stable isotope substitution. *E. coli* strain rUTI2 was grown in M63 media (top trace), with ^15^N-ammonium as the nitrogen source (middle trace), and with ^13^C-glycerol as the carbon source (bottom trace). The ^13^C-labeled forms are used as internal standards for mass spectrometric quantification in this study.

rUTI2 was chosen as a model strain to develop a quantitative metabolomic approach because it produced all four known *E. coli* siderophores. Thus, in iron-poor culture supernatants of strain rUTI2 we identified chromatography peaks corresponding to the [M+H]+ ions of aerobactin (14), salmochelin (15), and enterobactin (16), and the [M−2H+Fe(III)]+ ion of ferric yersiniabactin (17). These siderophore peaks elute from a reversed phase column in the order reported previously (19). Confirmatory structural information was available by comparing the *m/z* difference between the [M+H]^+^ of salmochelin and its precursor, enterobactin. The salmochelin [M+H]^+^ is 342 m/z units greater (Fig. 2), consistent with enterobactin di-*C*-glucosylation and trilactone hydrolysis catalyzed by IroB and IroE, respectively (20, 21).

To further confirm the identity of presumptive siderophore ions, rUTI2 was grown in defined minimal media in which ^13^C_3_-glycerol or ^15^N-ammonium sulfate were substituted for the unlabeled compounds. This heavy isotope labeling strategy resulted in mass shifts for each ion peak based on the number of carbons or nitrogens in their empiric formulae (Fig. 2). Labeling efficiency was high and no unlabeled siderophores or M+1 or M+2 carbon isotope distributions were observed for the most abundant ^13^C-labeled enterobactin, salmochelin, and aerobactin ions. The prominent M+1 and M+2 ions uniquely present in the ^13^C-labeled ferric yersiniabactin spectrum are consistent with the presence of iron and sulfur in this species. In this sample, the presence of a monoisotopic ^13^C ion clearly differentiates the M+1 ion from ^57^Fe (base peak contains ^56^Fe) and the M+2 ion from ^34^S (base peak contains ^32^S). After mixing labeled and unlabeled supernatants, the labeled and unlabeled siderophore ions all co-eluted, consistent with their expected identical structures. This isotope labeling technique provides both structural confirmation and a source of stable isotope labeled internal standards for MS-based quantification.

MS/MS fragmentations were also studied for further structural confirmation using strain rUTI2 (Table S1). The enterobactin [M+H]^+^ at *m/z* 670 fragmented predominantly at the ester bonds to yield dihydroxybenzoyl serine monomer (*m/z* 224) and dimer (*m/z* 446) as previously reported [15]. In contrast, MS/MS spectra of salmochelin gave the distinct lower molecular weight species expected from fragmentation within the glucose moieties, a finding supported by unchanged neutral losses from ^15^N-labeled products. Consistent with the hallmark *C*-glucosylation in salmochelin, we observed no loss of glucose (neutral loss of *m/z* 162) as is typically seen with O- or N-linked sugars. The aerobactin [M+H]^+^ at *m/z* 565 fragmented to give the neutral losses of water (*m/z* 547) and HCOOH (*m/z* 519) of a multiply hydroxylated and carboxylated compound. MS/MS of ferric yersiniabactin [M−2H+Fe(III)]^+^ gave a complex spectrum, as expected from a heterocyclic compound, that included the prominent *m/z* 489 peak observed in previous MALDI spectra [16]. Together, these ion fragmentation patterns were consistent with the known structures of the corresponding siderophores. These MS/MS fragmentations were used to quantify siderophores in a multiplexed LC-MS/MS assay.

### Effect of mutations in siderophore biosynthetic genes on metabolomic profile

**Figure 3:**
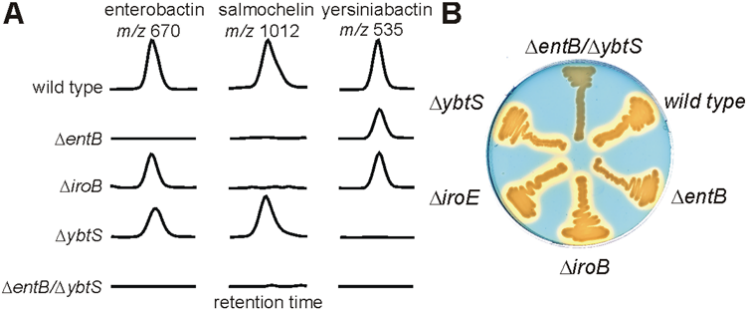
Siderophore production by the model uropathogen UTI89 and selected mutants. A) MS/MS chromatograms showing siderophore production in wild type UTI89 and strains with deletions in specific siderophore biosynthesis genes (Δ*entB*, Δ*iroB*, Δ*ybtS*, Δ*entB*/Δ*ybtS*). Chromatograms are shown at the retention times for enterobactin (*m/z* 670), salmochelin (*m/z* 1012), and ferric yersiniabactin (*m/z* 535). For each column, the vertical scale is a fixed fraction of the corresponding ^13^C internal standard peak height, allowing comparison between samples. B) Chrome azurol S (CAS) plate upon which UTI89 strains have been streaked for overnight growth. A yellow halo is produced around siderophore-secreting bacteria. In UTI89, a double mutant (Δ*entB*/Δ*ybtS*) is required to specifically abolish siderophore production.

The sequenced model uropathogen UTI89 was observed to produce enterobactin, salmochelin, and yersiniabactin. To validate the stable isotope dilution LC-MS/MS metabolomic assay, we analyzed UTI89 strains with deletion mutations in selected siderophore biosynthetic genes (Fig. 3A). Ions corresponding to the catecholate siderophores enterobactin and salmochelin were absent in an *entB* [17] mutant while yersiniabactin production was preserved. Conversely, the *ybtS* [18] mutant produced enterobactin and salmochelin but not yersiniabactin. Because *ybtS* encodes a salicylate synthase, yersiniabactin expression could be restored in UTI89Δ*ybtS* by growth in the presence of exogenous 0.3 mM sodium salicylate (data not shown). Selective loss of salmochelin was observed with deletion of *iroB*, which forms C-glucose bonds with enterobactin [19]. These findings show that metabolomic profiling is sensitive to alterations in siderophore biosynthetic pathways.

### Accountability of total siderophore activity using genetics and metabolomics

Single deletion mutants in the siderophore biosynthetic pathways described above (*entB*, *ybtS*, *iroB*) remained positive for siderophore production by the chrome azurol S plate assay based on blue-to-yellow transformation surrounding streaked colonies [20] (Fig. 3B). To determine whether enterobactin, salmochelin, and yersiniabactin together correspond to the total siderophore activity expressed by UTI89, we constructed a UTI89Δ*entB*Δ*ybtS* double mutant which was predicted to selectively abolish synthesis of all known siderophores in UTI89. Metabolomic profiling of UTI89Δ*entB*Δ*ybtS* confirmed the absence of all three siderophores in this mutant and the chrome azurol S assay revealed unchanged colony growth without siderophore production. Thus, for UTI89, total siderophore activity is accountable using this metabolomic analysis as confirmed using a combined biochemical, genetic, and chemical approach. Unlike the K12 *E. coli* strains described previously [21],[22], a single mutation within the enterobactin gene cluster is insufficient to abolish siderophore production from UTI89.

### Siderophore receptor genotype does not predict expression of the corresponding siderophore

To compare siderophore expression phenotype to bacterial genotype, we examined siderophore production by three fully sequenced *E. coli* strains MG1655 [23], UTI89 [6], and CFT073 [24] and in our panel of 18 previously genotyped clinical *E. coli* urinary isolates [14] described above (Table 2). The yersiniabactin receptor (*fyuA*) and aerobactin receptor (*iutA*) genotypes were known for all 21 strains. Of the 16 *fyuA*-positive strains, three pyelonephritic strains, CFT073, *pyelo1*, and *pyelo3* produced no detectable yersiniabactin despite producing other siderophores. Of the 8 *iutA*-positive strains, two, *rUTI4* and *pyelo3*, produced no detectable aerobactin while still producing other siderophores. The inability of CFT073 to synthesize yersiniabactin is presumably due to mutations that have been identified within essential yersiniabactin biosynthetic genes in this strain [25]. Thus, receptor genotype does not consistently predict the cohort of siderophores that are presented by an organism in response to low-iron conditions.

### Recovery of same-patient rectal-urinary *E. coli* strain pairs

To determine whether urinary *E. coli* strains exhibited preferential siderophore expression when compared to distinct, coincident rectal strains, we collected a new set of *E. coli* strains from 18 recurrent UTI patients and we PFGE-typed the isolates (this is a distinct set of isolates from those described in Table 2). From this collection, we identified 13 patients in whom distinct, coincident urinary and rectal PFGE types associated with a UTI were recovered. Clinical characteristics of these 13 patients are described in Table 3. Coincident rectal strains were defined as those isolated from rectal swabs obtained up to one month prior to isolation of a distinct urinary PFGE type. Thus, for this study 14 urinary and 16 rectal PFGE types were chosen from this set of 13 patients. Similarity analysis based on PFGE typing revealed marked diversity (Fig. S1). Two patients yielded isolates with the same PFGE type (Patients 34 and 50, Table 4), one from urine and the other from the rectum. All 30 urinary and rectal strains from these 13 patients exhibited similar growth patterns under iron-limited, minimal media conditions [mean difference 0.007; 2-tailed *p* = 0.8273], indicating that observed metabolite differences were not related to differences in growth density.

**Table 3:** Patient characteristics for paired strain study.

Variable	Case-Patients
	(n = 13)
Age, %	
18–30 y	85%
31–49 y	15%
Mean age±SE, *y*	24.2±1.5
Previous UTIs, %	
0	0%
1–4	46%
≥5	54%
#UTIs during study	
1	1
2	7
3	5

**Table 4:** Patient PFGE types, siderophore expression, and genotypes related to source and UTI event.

patient #	mst #	source	UTI #	yersiniabactin		salmochelin		aerobactin	
				MS	*fyuA* PCR	MS	*iroN* PCR	MS	*iutA* PCR
*2*	*0288*	U	1	−	−	+	+	−	−
	*3103*	R	1	−	−	−	−	−	−
*11*	*0380*	U	1	−	−	−	−	−	−
	*1343*	R	1	−	−	−	−	−	−
	*1995*	R	1	−	−	−	−	−	−
*13*	*0355*	U	1,2,3	+	+	+	+	−	−
	*10727*	R	1,2,3	+	+	+	+	−	−
*17*	*0349*	U	1	+	+	−	−	−	+
	*0396*	R	1	−	−	−	−	−	−
	*2355*	R	1	−	−	−	−	−	+
*34*	*6746*	U	1	+	+	+	+	−	−
	*8047*	R	1	+	+	+	+	−	−
*35*	*6747*	U	1	+	+	+	+	−	−
	*10760*	R	1	+	+	−	−	−	−
*50*	*8049*	U	1	+	+	+	+	−	−
	*6746*	R	1	+	+	+	+	−	−
*54*	*1109*	U	1,2,3	+	+	−	−	−	−
	*10917*	R	1,2	−	−	−	−	−	−
	*10920*	R	3	−	−	−	−	−	−
	*10921*	R	3	+	+	−	−	−	−
*56*	*1126*	U	1,2	+	+	+	+	−	+
	*1110*	R	1,2	−	+	−	−	+	+
*68*	*6748*	U	1	+	+	+	+	+	+
	*10932*	R	1	−	−	−	−	−	+
*72*	*6745*	U	1,2	−	−	−	−	−	−
	*10775*	R	1,2	−	−	−	−	−	−
*94*	*5790*	U	1	+	+	−	+	−	−
	*9282*	R	1	−	+	−	−	+	+
	*9282*	U	2	−	+	−	−	+	+
	*13579*	R	2	−	−	−	−	−	−
*110*	*0378a*	U	1	+	+	−	−	−	+
	*13590*	R	1	+	+	−	−	+	+

The presence or absence of siderophore production as determined by mass spectrometry (MS) is indicated with the presence or absence of the corresponding siderophore receptor genotype as determined by PCR.

mst, multilocus sequence type; R, rectal; U, urinary; UTI, urinary tract infection; MS, mass spectrometry.

### Differences in siderophore production between same-patient rectal and urinary strains

**Figure 4:**
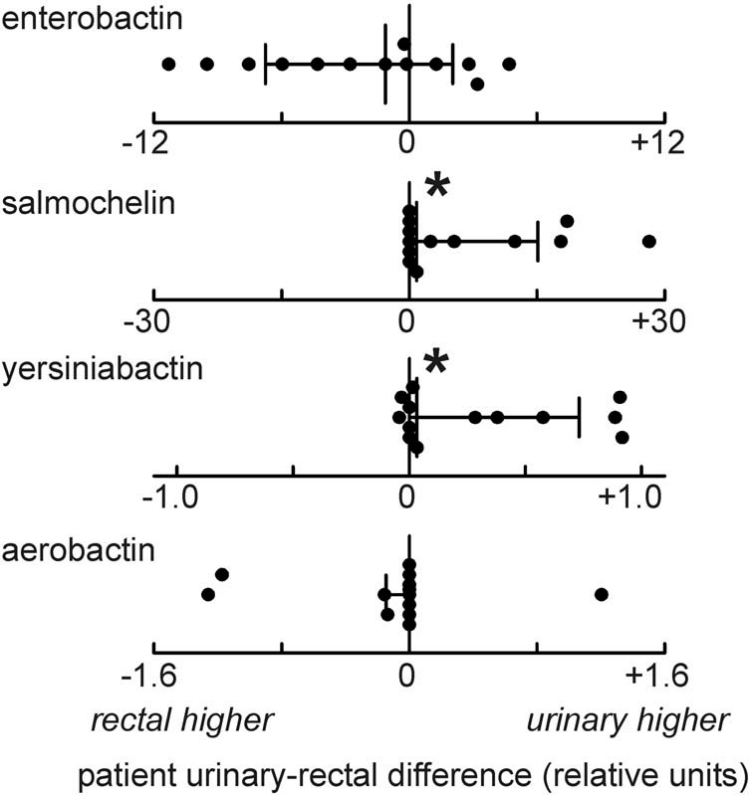
Differences in siderophore production between single-patient urinary and rectal isolates. Each data point is the difference in siderophore production between coincident urinary and rectal strains recovered from an individual with UTI. These differences were determined by stable isotope dilution mass spectrometry and expressed in reference strain equivalents. The median and interquartile range is depicted for each siderophore. *Medians differs significantly (*p*<0.05) from zero.

To determine if quantitative and qualitative differences in the siderophore metabolome distinguish urinary from rectal isolates in this set of 13 patients, we used quantitative metabolomic profiling and then compared these results to a genotypic analysis. In the metabolomic analysis, we measured differences between coincident urinary and rectal strains within individual patients. For each patient, the quantity of each siderophore produced by rectal strains was subtracted from the quantity produced by the coincident urinary strains to yield a difference (Fig. 4). In the four patients from whom multiple coincident urinary and rectal strains were recovered, the mean difference in siderophore production is reported. This analysis revealed significantly (*p*<0.05) higher salmochelin and yersiniabactin production among urinary strains. It was also notable that we found no instances in which the rectal-only strain produced yersiniabactin or salmochelin while the urine strain did not. Furthermore, urinary strains always produced more salmochelin, even when non-urinary strains also expressed salmochelin (n = 3) suggesting that salmochelin biosynthesis was more active in urinary strains. Among all urinary strains in this study, prevalence was in the order enterobactin (100%), yersiniabactin (71%), salmochelin (50%) and aerobactin (14%). These data show that, while all strains made enterobactin, a biosynthetically active *Yersinia* HPI and *iroA* cassette were common among urinary isolates in this population and that production of these siderophores may have a clinically evident impact on UTI recurrence.

### Comparison between siderophore expression and siderophore receptor genotype

The siderophore expression analysis of the rectal and urinary strains from the 13 patients described above was compared to PCR genotyping using established PCR primers for siderophore receptor genes (*fyuA* for yersiniabactin, *iroN* for salmochelin, *iutA* for aerobactin) [8],[26] (Table 4). Previous genotypic data suggesting that enterobactin genes are conserved among *E. coli* was supported by product analysis, which revealed enterobactin production by all patient isolates. For yersiniabactin and salmochelin, the siderophore receptor genotyping analysis had the limitation of being unable to discern strain differences when both a rectal and urinary strain pair were genotype positive. This was a frequent occurrence. In the ten patients from whom at least one *fyuA*+ strain was recovered, product analysis revealed rectal-urinary differences in yersiniabactin production in all ten, while genotyping predicted differences in four. Similarly, in the eight patients from whom at least one *iroN*+ strain was recovered, product analysis revealed differential yersiniabactin production in seven, while genotype predicted differential expression in four. Among *fyuA*− or *iroN*-positive strain pairs, mean and median yersiniabactin or salmochelin production remained higher among the urinary strains.

For aerobactin, differences between *iutA* PCR and product analysis were even more striking. Aerobactin production was only detected in four of nine *iutA*+ strains. As a result, differences in siderophore production predicted by genotype were changed by product analysis in four patients. Agreement between the two methods occurred in one patient with an *iutA*+ strain and in the seven patients with *iutA*− strains that produced no detectable aerobactin. These data comparing PCR genotyping to product analysis demonstrate that differences in biosynthetic activity are not solely a reflection of the presence or absence of siderophore gene loci.

### Salmochelin and yersiniabactin expression are correlated

Yersiniabactin was the most prevelant non-enterobactin siderophore and strikingly, was co-expressed in 90% of the strains that expressed salmochelin. This was the most frequent co-association of any of the non-enterobactin siderophores (*p* = 0.006) and was mirrored by a significant association between *fyuA* and *iroN* positivity (*p* = *0.008*). Salmochelin and yersiniabactin co-expression was seen more often among patient urinary (6/13 (46%)) than rectal (3/13 (23%)) strains, although this trend did not reach statistical significance (*p* = 0.18). Thus, salmochelin expression tended to occur in addition to yersiniabactin expression and co-expression was a common feature among urinary strains in this population. These data raise the possibility that these two siderophore types exhibit complementary activities.

### Enterobactin linearization is an additional virulence activity of strains with an active *iroA* cassette and is consistent with IroE esterase activity

**Figure 5:**
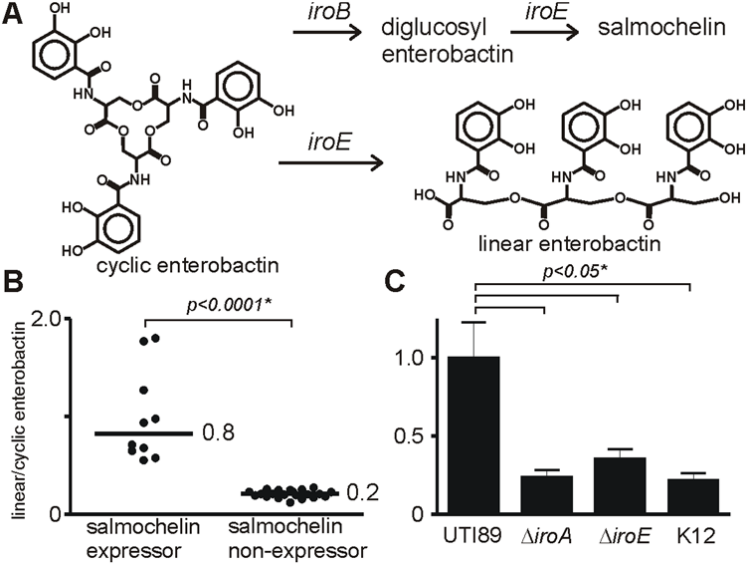
In addition to directing salmochelin biosynthesis, the *iroA* cluster also increases production of linear enterobactin, a candidate virulence-associated siderophore. A) Cyclic enterobactin is converted into a linear form by ester bond hydrolysis. B) Clinical isolates that produce salmochelin (+salmochelin) exhibit greater enterobactin linearization than salmochelin nonproducers (-salmochelin). C) Removal of the entire *iroA* cluster (*iroBCDEN*) significantly reduced enterobactin linearization. A similar reduction in enterobactin linearization is seen in an *iroE* mutant. Enterobactin linearization in MG1655, a K12 strain without the *iroA* cluster, was comparable to that seen in these mutants. These findings support the esterase IroE as an enterobactin linearizing enzyme *in vivo*.

Clinical isolates were noted to produce varying amounts of linearized enterobactin, which is distinguished by a distinct retention time and an ion at *m/z* 688, consistent with hydrolysis (+18 amu) of a single ester bond (Fig. 5A). While somewhat slower at scavenging iron than enterobactin, it has been proposed that linear enterobactin is better suited to avoid sequestration by hydrophobic surfaces [27]. Enterobactin linearization was quantified by the precursor/product relation:
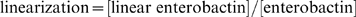



Salmochelin expressors exhibited over threefold higher enterobactin linearization than non-expressor strains (Fig. 5B). To explore genetic correlates of this relationship, we examined enterobactin linearization among mutants in the *iroA* gene cluster in UTI89 (Fig. 5C). Mutants containing a deletion of the *iroA* gene cluster were unable to express salmochelin and exhibited a decrease in enterobactin linearization compared to the wild type control. Similar low levels of linear enterobactin were observed in clinical isolates that did not express salmochelin. To determine whether this linearizing activity was attributable to the esterase IroE [28], we examined enterobactin linearization in an *iroE* mutant. Linearization by the *iroE* mutant was decreased to the same levels as in the K12 strain MG1655, which lacks all of the *iroA* genes. The remaining baseline level of linear enterobactin in the absence of the *iroA* genes has been observed previously and may derive from premature release during biosynthesis [29], cleavage of ferric enterobactin by the enterobactin esterase Fes, or spontaneous ester bond hydrolysis. Together, these data show that, in addition to synthesizing salmochelin, *iroA* also directs enterobactin linearization through the action of the esterase IroE to produce linear enterobactin, a siderophore that may be better suited to iron-scavenging during infection.

## Discussion

We have used a combined chemical, genetic, and patient-oriented approach to examine clinical correlates of siderophore production among human *E. coli* isolates associated with recurrent urinary tract infection. Development of a quantitative metabolomic approach allowed assessment of the complex multiprotein biosynthetic pathways for siderophores rather than inferring these activities from genotype or transcription analysis alone. PCR genotyping of a single siderophore system gene was not an efficient predictor of siderophore production during iron-restricted growth. Thus, the quantitative metabolomic approach was used to determine whether successful uropathogens exhibit systematic differences from coexisting rectal and urinary strains in individual patients. Rather than comparing strains across a population, we examined strain differences within individual patients in order to compare each urinary strain to a more valid reference population.

This study compares siderophore production between coexisting bacterial strains associated with urinary disease and gut colonization. Since uropathogens are thought to arise from the gut flora, comparison of these populations should represent the most valid study design. The dichotomy between commensalism and pathogenicity is a common theme among bacteria and is particularly compelling in the case of *E. coli* urinary tract infections. Siderophore expression has been linked to virulence and here we show that yersiniabactin and salmochelin were the most common non-enterobactin siderophores associated with UTI recurrence in this typical young female population. Notably, yersiniabactin synthesis was observed in almost all strains that expressed salmochelin. In this study population of young women with recurrent UTI, urinary strains produced greater amounts of two siderophores, yersiniabactin and salmochelin and co-expression of both of these siderophores was common.

In this study, quantitative product analysis provided information beyond conventional siderophore receptor genotyping in two circumstances: 1) when a genotype-positive strain was unable to produce detectable levels of the corresponding siderophore and 2) when siderophore production differed significantly between genotype-positive strain pairs. Ten of the thirteen patients in this study yielded at least one strain pair in which either or both of these circumstances was operative. Deficient or enhanced siderophore biosynthesis may arise in multiple environmental contexts. Pathogenic bacteria may benefit from increased production of siderophores that are better adapted to the infection microenvironment, as may be the case with salmochelin and yersiniabactin. Alternatively, bacterial strains in polymicrobial communities may benefit from inactivated siderophore production if they retain the ability to “steal” ferric siderophores produced by a neighbor, thereby avoiding the metabolic cost of siderophore biosynthesis [30],[31]. Thus, extrapolation of siderophore receptor genotype to biosynthetic phenotype is inefficient, often inaccurate and suggests that optimization of siderophore biosynthesis may occur in pathogenic strains.

Enterobactin or aerobactin production was not preferentially associated with urinary strains in this population. The lack of increased enterobactin production among urinary strains suggests that qualitative shifts in siderophore type may be more conducive to uropathogenesis than a quantitative shift in enterobactin production. Although *iutA* positivity among pathogenic strains is often used to conclude that aerobactin is an important virulence factor, we did not observe preferential expression of this siderophore when urinary and rectal isolates were compared. The sample size may not have allowed us to discern preferential aerobactin production in this population. Alternatively, *iutA*-positive clinical isolates might exhibit urinary virulence properties that are unrelated to aerobactin production.

**Figure 6:**
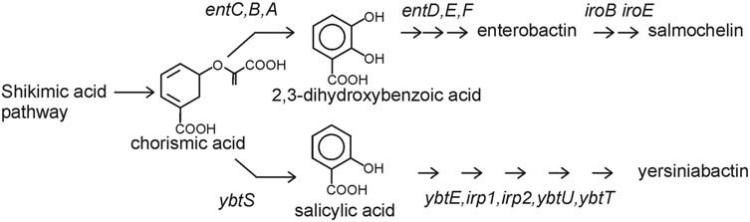
Biosynthesis of siderophores associated with recurrent UTI depends on chorismic acid utilization.

These results suggest that yersiniabactin and salmochelin expression may facilitate infection of the human urinary tract. This effect is not absolute, as there are urinary strains in this study that express neither siderophore. Although rectal isolates in this and other studies [32] have been observed to produce yersiniabactin and/or salmochelin, the impact of these siderophores upon fitness for gut colonization is unclear. The relatively high prevalence of yersiniabactin and salmochelin expression among urinary pathogens (Table 5), makes these nonessential metabolic pathways intriguing prospects for virulence-targeted therapies. Interestingly, the yersiniabactin and salmochelin biosynthetic pathways converge at chorismic acid, where each pathway uses related enzymes to synthesize the aromatic precursors 2-hydroxybenzoic (salicylic) acid and 2,3-dihydroxybenzoic acid (Fig. 6). Targeting either or both of these initial points in siderophore biosynthesis may represent a promising target for anti-virulence drug discovery or design.

**Table 5:** Prevalence of detectable siderophore expression among different PFGE types in this study according to source.

siderophore expressed	urinary source (n = 14)	rectal source (n = 16)
enterobactin	100% (14)	100% (14)
yersiniabactin	71% (10)	31% (5)
salmochelin	50% (7)	13% (2)
aerobactin	14% (2)	25% (4)

The number of strains in each category is indicated in parentheses.

## Materials and Methods

### Ethics statement

This study was conducted according to the principles expressed in the Declaration of Helsinki. The study was approved by the Institutional Review Board of the University of Washington. All patients provided written informed consent for the collection of samples and subsequent analysis.

### Bacterial strains and cultivation

To examine siderophore production in liquid culture, previously published conditions were used [33]. 3 hour cultures of *E. coli* grown in LB broth were diluted 1∶100 into M63 medium containing 0.2% glycerol and 10 mg/mL niacin and incubated for 18 h at 37 C in a rotary shaker.

### Deletion strain construction

Deletion mutations were made using the red recombinase method, as previously described, using pKD4 or pKD13 as a template and the primers as listed in Table S2. [34],[35] PCR was performed with flanking primers to confirm the appropriate deletions. Antibiotic insertions were removed by transforming the mutant strains with pCP20 [36] expressing the FLP recombinase. The resultant strains subsequently had no additional antibiotic resistance compared with the parental wt strain.

### Liquid chromatography-mass spectrometry (LC-MS)

The mass spectrometer used for the studies was a Thermo-Finnigan LCQ Deca (San Jose, CA) coupled to a Waters CapLC (Waters MA) equipped with a Vydac C18 MS column (0.3×150 mm). The flow rate was 6 ul/min with a gradient as follows: Solvent A (0.1% formic acid) was held constant at 95% and solvent B (80% acetonitrile in 0.1% formic acid) was held constant at 5% for 5 minutes, increased to 44% B in the next 60 minutes, and then to 95% B in the next 20 minutes. All data was collected in a positive mode. The spray voltage on the mass spectrometer was held constant at 4.5 K and the capillary temperature was 200°C. For CID experiments helium was used as the collision gas with the collision energy set to 32% of the maximum (∼5 eV). The isolation width was 3 amu. Quantitation was carried out in the SRM mode using ^13^C labeled standards as described above. Data was collected in the positive centroid mode. Ions were monitored with a window of +/−0.5 amu.

### Siderophore extraction

0.1 M ferric chloride was added to cell supernatants to a final concentration of 3.75 mM. After a 15 minute room temperature incubation the precipitate was removed by centrifugation. The supernatant was applied to a column packed with 200 uL of DEAE slurry [33]. The loaded column was washed with 0.5 mL of water and siderophores were eluted with 3 mL of 7.5 M ammonium formate adjusted to pH 3.6 with 7.5 M formic acid. The DEAE eluate was further purified and desalted by application to a Chrom P solid phase extraction column (250 mg, Supelco). The loaded column was washed with 2 mL of 0.1% formic acid in 10% acetonitrile. Siderophores were eluted following application of 2 mL of 0.1% formic acid in 80% acetonitrile. The eluate was then concentrated to 100–200 uL final volume in a centrifugal evaporator for MS analysis.

### Preparation of ^13^C-labeled internal standards

Internal standards were produced by rUTI2, a clinical urine isolate found to express all four known *E. coli* siderophore types, or UTI89. Strains were each grown for 3 hours in LB broth, which was subsequently inoculated 1∶100 into M63 medium containing 0.2% ^13^C_3_-glycerol (99+ atom %, Isotec), and 10 mg/mL niacin and incubated for 18 h at 37 C in a rotary shaker. Cells were removed by centrifugation and a frozen stock of supernatant was kept for use as internal standard. Isotopic labeling was confirmed by LC-MS.

### Quantification of siderophores using stable isotope dilution mass spectrometry

Strains to be compared, along with the reference strain rUTI2, were prepared together on the same day using the same media, reagents, and internal standard. Siderophore quantities are expressed as reference strain equivalents determined through the stable isotope dilution method. ^13^C-labeled internal standard was added 1∶1 to each clarified culture supernatant and mixed prior to siderophore extraction. Siderophore extracts subject to comparison were then prepared and analyzed by LC-MS/MS using the parent and daughter ions described above and listed in Table S1. Each siderophore type was first quantified by determining the ratio of the analyte peak to the co-eluting ^13^C-labeled internal standard peak. These peak area ratios were then converted to molar ratios by comparison to standard curves generated by mixing known ratios of unlabeled and labeled rUTI2 supernatants. Siderophore quantities were expressed as rUTI2 equivalents by normalizing each molar ratio to that observed for strain rUTI2 under identical culture conditions.

### PCR analysis

Isolates were grown to log phase on 5 ml LB medium. Primer combinations FyuA f'–FyuA r (880 bp product)/AerJ f–AerJ r (300 bp)/IRONEC-F–IRONEC-R (665 bp) were used for amplification of *fyuA*, *iutA*, and *iroN* genes, respectively [12],[37] Amplification reactions were carried out individually in a Bio-Rad MyCycler instrument using 5 µl of heat-inactivated culture and 35 cycles of 95×30″/57×30″/72×60″.

### Patients and strains

Patients presenting with UTI were enrolled and monitored prospectively as described previously [14]. Rectal specimens were collected during clinic visits using a sterile, rayon-tipped swab and transported to the laboratory in Amies Medium (BBL™ CultureSwab™ Plus, Becton, Dickinson). To avoid inclusion of rectal strains that may have been introduced after the UTI event or that may have been only transiently present [38],[39] prior to the UTI event, rectal strains were excluded if they were recovered after or >30 days before the urinary isolate.

### Statistical analysis

Statistics and graphs were generated using GraphPad Prism 4. For groupwise comparisons of siderophore production, the Mann-Whitney U Test was performed. Analyses of paired strain differences in siderophore production were performed using the Wilcoxon signed rank test for significance. For analysis of stationary phase density, the data passed the F test for equal variances and the *t* test was used to compare urinary versus rectal strain growth as well as growth differences between paired strains. Categorical data was analyzed using Fisher's exact test.

## Supporting Information

Figure S1 Dendrogram, PFGE patterns, siderophore status and hemolytic properties of patient strains collected for comparison of urinary strains with coexisting rectal strains.
(4.89 MB TIF)

Table S1 CID fragmentations used to identify and quantify siderophores by LC-MS/MS.
(0.03 MB DOC)

Table S2 Primers used in construction of UTI89 mutants.
(0.02 MB DOC)
