# Supplementary figures and images for: Quantitative Metabolomics Reveals an Epigenetic Blueprint for Iron Acquisition in Uropathogenic Escherichia coli
Source: PLoS Pathog. 2009 Feb 20;5(2):e1000305. doi: 10.1371/journal.ppat.1000305 (PMC2637984; doi:10.1371/journal.ppat.1000305)

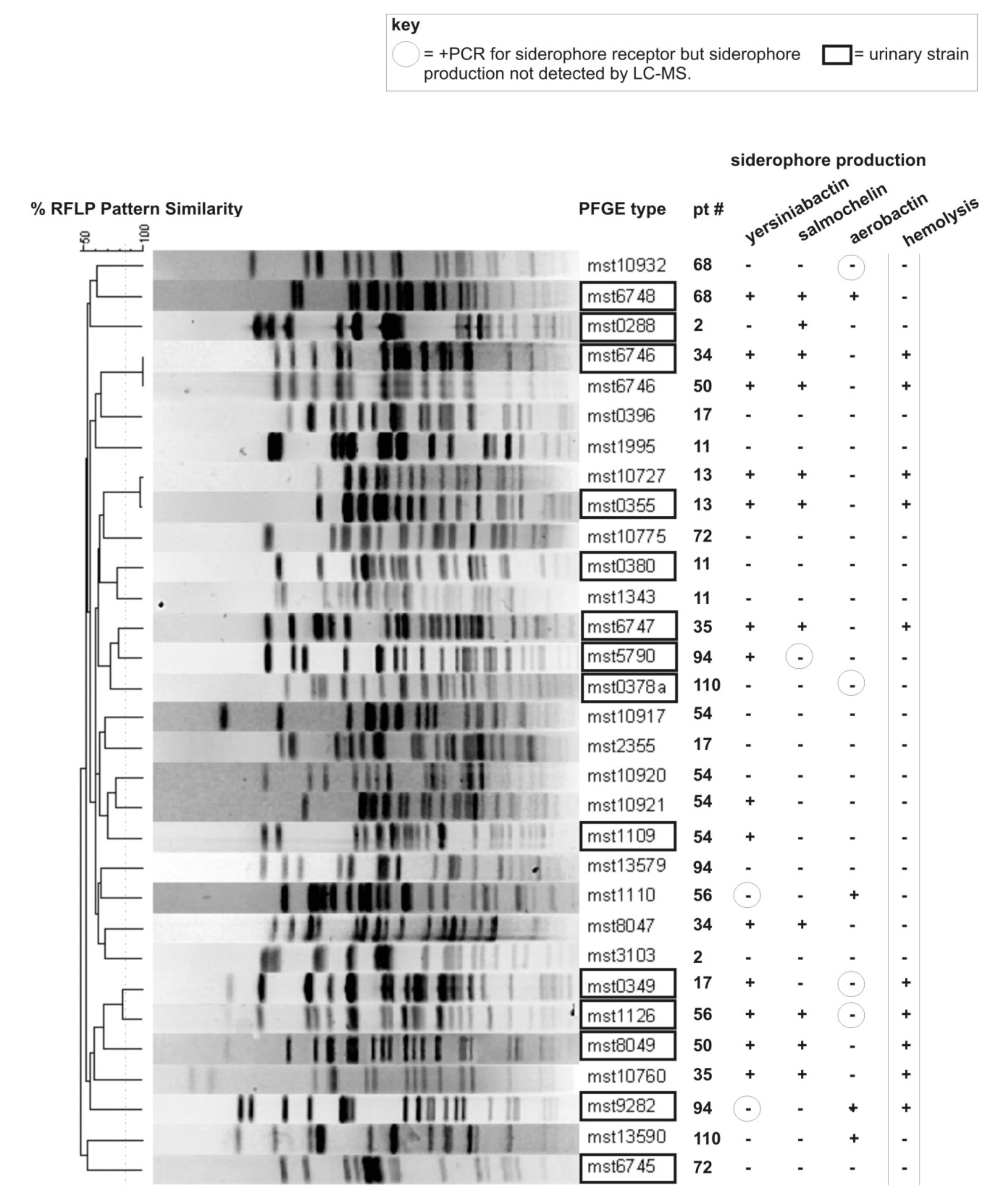

Supplement: Figure S1 — Dendrogram, PFGE patterns, siderophore status and hemolytic properties of patient strains collected for comparison of urinary strains with coexisting rectal strains. (4.89 MB TIF) [file ppat.1000305.s001.tif]
