# Supplementary material for: Quantitative Metabolomics Reveals an Epigenetic Blueprint for Iron Acquisition in Uropathogenic Escherichia coli
Source: PLoS Pathog. 2009 Feb 20;5(2):e1000305. doi: 10.1371/journal.ppat.1000305 (PMC2637984; doi:10.1371/journal.ppat.1000305)
Supplement: Table S1 — CID fragmentations used to identify and quantify siderophores by LC-MS/MS. (0.03 MB DOC) [file ppat.1000305.s002.doc]

**Supplementary Table 1**. *E. coli* siderophores monitored by LC-MS/MS.

| Siderophore | ^12^C *m/z* | ^13^C *m/z* |
| --- | --- | --- |
| enterobactin | 670 →224,447 | 700 →234,467 |
| linear enterobactin | 688 →224,447, 465 | 700 →234,467, 485 |
| salmochelin | 1012 →838,874,928,940,958 | 1054 →873,912,969,982,1000 |
| yersiniabactin | 535 →188,294,302,348,464,489 | 556 →196,304,314,361,482,509 |
| aerobactin | 565 →519,529,547 | 587 →540,551,569 |
