# Supplementary material for: Quantitative Metabolomics Reveals an Epigenetic Blueprint for Iron Acquisition in Uropathogenic Escherichia coli
Source: PLoS Pathog. 2009 Feb 20;5(2):e1000305. doi: 10.1371/journal.ppat.1000305 (PMC2637984; doi:10.1371/journal.ppat.1000305)
Supplement: Table S2 — Primers used in construction of UTI89 mutants. (0.02 MB DOC) [file ppat.1000305.s003.doc]

**Supplementary Table 2.** Primers used in construction of UTI89 mutants.

***entB***

left;ATGGCTATTCCAAAATTACAGGCTTACGCACTGCCGGAGTCTCATGATATGTGTAGGCTGGAGCTGCTTC

right;GAGTAAACGCTTGATCAAAACCTGTAACTTTCGCCCCCGCCTCAACAAATATTCCGGGGATCCGTCGACC

***iroB***

left;CGCATTGTTATGGATGCATGACTCCTGGATGGGTGCTGGCATATGATTATCATATGAATATCCTCCTTAG

right;TGCGTCGACTGCCTGATTTAGATCGTCAAGCGGAGAGGGATTTTCTCATGGTGTAGGCTGGAGCTGCTTC

***iroE***

left;TTAGTGGCTTAACTCATGACAACCTGCTGTGTAATTTGCGTTTTCACCACGTGTAGGCTGGAGCTGCTTC

right;GGAAAAGGAATAATCATCAGATGTATGCCCGCGAGTATCGCTCAACACGCCATATGAATATCCTCCTTAG

***iroA***

left;ATGAGAATTAACAAAATCCTCTGGTCGCTAACTGTGCTCCTGGTTGGGTTGTGTAGGCTGGAGCTGCTTC

right;GTGCGTCGACTGCCTGATTTAGATCGTCAAGCGGAGAGGGATTTTCTCATCATATGAATATCCTCCTTAG

***ybtS***

left;AGGTTAGAAAACAGTTACTCCTACACCATTAAATAGGGCGCAATGCTCGCGTGTAGGCTGGAGCTGCTTC

right;GCTGCATTCGGCAAGAGAACTGATGACAAGCGCAATTGATTGATCCGGGAATTCCGGGGATCCGTCGACC
